# PHRF-RTDETR: a lightweight weed detection method for upland rice based on RT-DETR

**DOI:** 10.3389/fpls.2025.1556275

**Published:** 2025-06-24

**Authors:** Xianjin Jin, Jinheng Zhang, Fei Wang, Mengyan Zhao, Yunshuang Wang, Jianping Yang, Jinfeng Wu, Bing Zhou

**Affiliations:** ^1^ College of Big Data, Yunnan Agricultural University, Kunming, China; ^2^ College of Science, Yunnan Agricultural University, Kunming, China; ^3^ College of Plant Protection, Yunnan Agricultural University, Kunming, China; ^4^ College of Food Science and Technology, Yunnan Agricultural University, Kunming, China

**Keywords:** weed detection, lightweight, PHRF-RTDETR, PGRNet, HiLo, RetC3, Focaler-WIoUv3

## Abstract

**Introduction:**

Weed poses a greater threat to rice yield and quality in upland environments compared to paddy fields. Effective weed detection is a critical prerequisite for intelligent weed control technologies. However, the current weed detection methods for upland rice often struggle to achieve a balance between accuracy and lightweight design, significantly hindering the practical application and widespread adoption of intelligent weeding technologies in real-world agricultural scenarios. To address this issue, we enhanced the baseline model RT-DETR and proposed a lightweight weed detection model for upland rice, named PHRF-RTDETR.

**Methods:**

First, we propose a novel lightweight backbone network, termed PGRNet, to replace the original computationally intensive feature extraction network in RT-DETR. Second, we integrate HiLo, a mechanism excluding parameter growth, into the AIFI module to enhance the model’s capability of capturing multi-frequency features. Furthermore, the RepC3 block is optimized by incorporating the RetBlock structure, resulting in RetC3, which effectively balances feature fusion and computational efficiency. Finally, the conventional GIoU loss is replaced with the Focaler-WIoUv3 loss function to significantly improve the model’s generalization performance.

**Results:**

The experimental results show that PHRF-RTDETR achieves precision, recall, mAP50, and mAP50:95 scores of 92%, 85.6%, 88.2%, and 76.6%, respectively, with all metrics deviating by less than 1.7 percentage points from the baseline model in upland rice weed detection. In terms of lightweight indicators, PHRF-RTDETR achieved reductions in floating-point operations, parameter count, and model size by 59.3%, 53.7%, and 53.9%, respectively, compared to the baseline model. Compared with the traditional target detection models of Faster R-CNN and SSD, YOLO series models, and RT-DETR series models, the PHRF-RTDETR model effectively balances lightweight and accuracy performance for weed detection in upland rice.

**Discussion:**

Overall, the PHRF-RTDETR model demonstrates potential for implementation in the detection modules of intelligent weeding robots for upland rice systems, offering dual benefits of reducing agricultural production costs through labor efficiency and contributing to improved food security in drought-prone regions.

## 1 Introduction

Rice (*Oryza sativa*) is one of the world’s three major food crops, with an annual output of approximately 800 million tons, accounting for a quarter of global grain production (Bai et al., 2023). It serves as the staple food for nearly 50% of the world’s population (Sun et al., 2022). Rice is classified into lowland and upland types (Xia et al., 2019), with the primary distinction based on the growing environment. Upland rice is cultivated in areas with limited water resources and unpredictable precipitation, relying on natural rainfall or limited irrigation, unlike lowland rice, which is grown in paddy fields. As climate change intensifies and water resources become scarcer, the area dedicated to upland rice cultivation in arid and semi-arid regions is expected to expand, while a reduction in paddy field area is projected, helping to alleviate pressure on global food security (Alagbo et al., 2022). However, a key challenge in upland rice cultivation is weed competition. Drylands inherently provide fewer resources for growth than paddy fields. Weeds compete with upland rice for these limited resources, severely hindering its growth and substantially reducing both yield and quality. Therefore, developing effective weed removal measures is critical to alleviate these constraints in upland rice cultivation systems.

Currently, there are four primary methods of weed removal: preventive, biological, chemical, and mechanical weeding (Hasan et al., 2021). Among these, the chemical and mechanical methods remain widely adopted in agricultural practice. Intelligent weeding robots, equipped with precise weed detection capabilities, can significantly enhance the removal efficiency through targeted herbicide application and physical elimination. Given the challenge of labor shortages in agricultural production (Yang et al., 2021), it is crucial to advance the detection technologies used in intelligent weeding robots.

Traditional machine learning methods for weed detection rely on features such as color, shape, vein pattern, size, and texture (Li et al., 2024). Sujaritha et al. (2017) extracted features from sugarcane leaf weeds using morphological operations and combined them with fuzzy real-time classification technology to distinguish the weeds. Ashraf and Kha (Ashraf and Khan, 2020) classified rice weed density images into three categories using two classification techniques. The first technique extracts texture features from grayscale co-occurrence matrices (GLCM) and applies radial basis functions (RBF) with support vector machines (SVM), achieving an accuracy of 73%. The second technique uses scale- and rotation-invariant moment features combined with a random forest classifier, achieving 86% accuracy. Dadashzadeh et al. (2020) extracted 302 features related to color, shape, and texture and then optimized an artificial neural network (ANN) for weed classification using particle swarm optimization (PSO) and the bee algorithm (BA). The results indicate that the accuracy of the ANN-BA classifier is 88.74% and 87.96%, respectively, for the two test sets. Bakhshipour and Jafari (2018) integrated Fourier descriptors with invariant moment features using SVM and ANN to classify common weeds in sugar beet fields and achieved high accuracy. The results show that the SVM classifier achieved an overall accuracy of 95%, while the ANN achieved 92.92%. While these methods focus on enhancing traditional machine vision techniques for weed detection and have yielded promising results, the convergence among weed species significantly limits the efficacy of unimodal feature analysis, compelling the use of multidimensional feature combinations. This paradigm introduces two fundamental constraints: (1) When processing large-scale, real-time data, these methods rely on time-consuming manual feature extraction, resulting in low efficiency. (2) These methods exhibit limited robustness, particularly in overcoming environmental challenges such as occlusion, clustering, and illumination changes.

In unstructured environments, deep learning outperforms traditional image processing techniques (Kamilaris and Prenafeta-Boldú, 2018) due to its ability to automatically extract features and handle high-dimensional data (Wang S. et al., 2024). Some researchers have applied convolutional neural network (CNN) architectures for weed detection—for example, Jiang et al. (2020) proposed a graph convolutional network (GCN) based on CNN features, achieving recognition accuracies of 97.80%, 99.37%, 98.93%, and 96.51% on four different weed datasets. This method outperformed AlexNet, VGG16, and ResNet-101. Jiang et al. (2019) used Inception-ResNet-v2 as the backbone of Faster R-CNN to detect crops and weeds in cotton fields, achieving F1 scores of 72.7% and 96.9% at IoU thresholds of “all” and 0.5, respectively. Lam et al. (2021) employed VGG-16 to classify drone images of *Rumex obtusifolius* weeds, achieving an overall accuracy of 92.1% and an F1 score of 78.7%. Zou et al. (2022) proposed an enhanced U-Net for segmenting wheat and weeds in images. Transfer learning was applied during training, resulting in a segmentation IoU of 88.98% and an average processing speed of 52 FPS on embedded devices.

Although classical convolutional networks have yielded promising results in weed detection, their real-time detection performance is inferior to that of YOLO. As a result, research has increasingly focused on YOLO-based algorithm. Wang et al. (2022) combined YOLOv5 with the Convolutional Block Attention Module (CBAM) to develop the YOLO-CBAM model for detecting invasive alien weeds, such as *Solanum rostratum* Dunal. After the multi-scale training, the accuracy of YOLO-CBAM increased from 83.54% to 90.36%, while the model size was 14.9 MB. Shao et al. (2023), addressing the suboptimal weed detection performance of lightweight YOLOv5s, introduced the Ghost, C3 Trans, and CBAM modules into the backbone network to improve feature extraction in complex environments. They also incorporated the Bi-directional Feature Pyramid Network (BiFPN) structure in the neck network for multi-scale feature fusion. Additionally, a scale-sensitive crossover loss function was applied to reduce redundant bounding boxes. The final GTCBS-YOLOv5s achieved 91.1% mAP for detecting six weed species in paddy fields, with a processing speed of 85.7 FPS. Based on the YOLOv4 model, Chen et al. (2022) incorporated the Squeeze-and-Excitation (SE) module as the logic component in the SPP layer and introduced an adaptive spatial feature fusion structure in the feature fusion layer. The F1 scores of the YOLO-SEAM model for sesame and weeds were 0.91 and 0.92, respectively. Hu et al. (2024) proposed the Multimodule-YOLOv7-L, a lightweight weed severity classification model for lettuce rows, by selecting a YOLOv7 model with a scale factor (τ = 0.5) and combining the Efficient Channel Attention (ECA) and CA mechanisms, along with the ELAN-B3 and DownC modules. The model achieved a precision of 97.5% and a model size of 18.4 MB, successfully balancing the lightweight design with effective weed severity detection.

In summary, deep learning has become a dominant methodology in weed detection. However, the current research faces two critical limitations: (1) weed detection technologies have been primarily optimized for paddy fields, leaving upland farming systems comparatively neglected. (2) While current object detection techniques achieve high accuracy, their large model sizes and substantial computational requirements hinder their practical deployment in agricultural environments. The PHRF-RTDETR model, a deep learning-based approach with enhanced end-to-end object detection capabilities, shows great promise for weed detection in upland rice fields. This model is suited to overcome the challenges associated with the environment of upland rice system and the intrinsic diversity of weeds, which collectively exacerbate detection difficulties. By maintaining high detection accuracy, this study proposes a lightweight adaptation of the RT-DETR model. The key contributions of this work are outlined as follows:

1. By integrating the design principles of partial convolution (PConv), group convolution (GConv), and residual structures, we propose a new lightweight module called PGRBlock, which is highly efficient and multi-scale. Building upon PGRBlock and conventional convolution techniques, we develop a novel, efficient, and multi-scale lightweight backbone network named PGRNet. This backbone network significantly reduces computational and storage costs while effectively extracting key features of upland rice weed.
2. Building on the AIFI module, the AIFI-HiLo structure was developed by integrating the HiLo attention mechanism excluding parameter growth, which can capture both high- and low-frequency features. This modification addresses the limitation of the original AIFI model, where the multi-head attention mechanism inadequately emphasized the frequency-specific characteristics of weed in upland rice.
3. To address the issue of RepC3’s inability to handle convolutional redundancy and effectively capture long-range features, the RetBlock structure is introduced, resulting in the development of the RetC3 module. This module successfully achieved a lightweight design while ensuring feature fusion for weed in upland rice.
4. Focaler-WIoUv3 was derived by combining WIoUv3 with Focaler-IoU, effectively addressing the limitation of the original GIoU loss function when handling overlapping target frames for weed in upland rice.

## 2 Materials and methods

### 2.1 Weed database for upland rice

#### 2.1.1 Data acquisition

**Figure 1:**
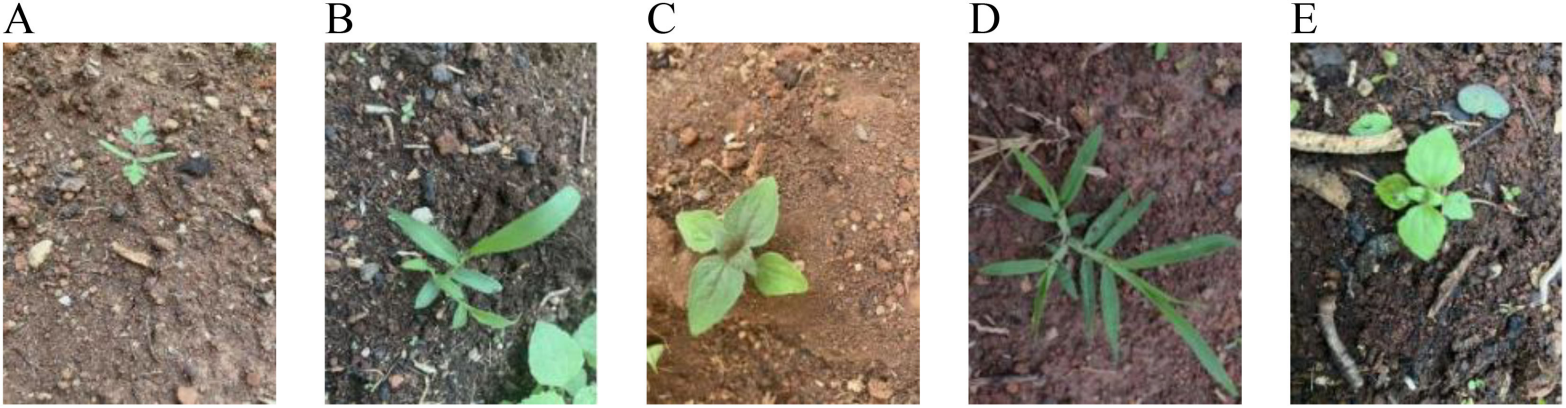
Examples of weed: **(A)** beggartick, **(B)** crabgrass, **(C)** *Galinsoga quadriradiata*, **(D)** goosegrass, and **(E)** tropic ageratum herb.

The weed images in this study were collected at the experimental base of Yunnan Agricultural University from July to August 2024. The specific shooting periods were daily from 9:00 to 12:00 in the morning and 16:00 to 19:00 in the afternoon to obtain weed image data at seedling and growth stages under different lighting conditions. The shooting equipment was an iPhone11, and the device’s built-in camera software was used for collection. The image resolution was uniformly adjusted to 640 × 640 pixels for input into the model for training. During constant-velocity image acquisition, a handheld camera was maintained at approximately 30 cm above ground level to replicate the near-ground perspective of a robotic weeder. The camera position remained unfixed to permit dynamic adjustment of the shooting angle, including both top-down and lateral views. To reflect the prime growth characteristics of weeds in the early stage, we collected the images of individual weeds, images of multiple weed communities, and images of weeds with mutual occlusion. The original dataset consists of 986 images representing five weed species: beggartick, crabgrass, *Galinsoga quadriradiata*, goosegrass, and tropic ageratum herb. Some sample images are shown in **Figure 1**.

#### 2.1.2 Data annotation

The training of the RT-DETR model follows a supervised learning approach, which requires labeled data to define class information and provide a basis for comparing each output with the true labeled data. Therefore, the weed dataset for upland rice was labeled accordingly.

First, the target categories were assigned labels 0, 1, 2, 3, and 4. Each weed target was then marked with a minimal bounding box to ensure that the area covered by the box is minimally affected by the background elements. The initial labeled data was saved in a text file format to meet the input requirements of the RT-DETR and YOLO family models. A Python script was then used to convert the text files into XML format, compatible with the SSD and Faster R-CNN models. Both file formats contain similar information, including category and bounding box details, but differ in their structure. In the text file, category information is represented by numerical values, while the bounding box is defined by the center coordinates, along with the relative height and width. The XML file uses English words to represent the categories, and the bounding box is specified by the X and Y coordinates of the top-left and bottom-right corners.

#### 2.1.3 Data demarcation and augmentation

Data augmentation techniques include random cropping, rotation, and blurring. Random cropping enables the model to learn features of weeds at various scales and positions, thereby improving sensitivity to local features and robustness to target location variations. Rotation operation facilitates the model’s recognition of weeds from different angles, particularly enhancing its adaptability to target orientation changes, which is crucial for distinguishing morphologically similar weeds. Blur processing simulates out-of-focus or suboptimal shooting conditions, allowing the model to learn weed characteristics from blurred or low-quality images, effectively improving its generalization capability in real-world environments. Collectively, these data augmentation strategies enhance the diversity of the weed dataset and simulate interference factors in real-world scenarios, significantly improving the model’s robustness and generalization ability, making it more suitable for practical upland rice field applications.

The original weed dataset in upland rice was partitioned into training, validation, and test sets with a ratio of 7:1:2. Data augmentation was then applied to expand the dataset. The distribution of the dataset before and after augmentation is shown in **Table 1**. Partitioning the data before augmentation is crucial to prevent data leakage. If data augmentation were applied to the entire dataset before splitting, the data in the test and validation sets may contain augmented samples from the training set. This would lead to issues such as evaluation bias, overfitting, and poor generalization (Wen et al., 2020).

**Table 1:** Demarcation and augmentation of weed dataset in upland rice.

Time	Type	Beggartick	Crabgrass	*Galinsoga quadriradiata*	Goosegrass	Tropic ageratum herb	Total
Before	Train	184	83	134	178	111	690
	Val	16	10	22	33	17	98
	Test	49	27	33	50	45	198
	Total	249	114	189	261	173	986
After	Train	708	427	505	674	436	2,750
	Val	59	51	80	122	68	380
	Test	182	123	131	199	180	815
	Total	949	601	716	995	684	3,945

### 2.2 Construction of lightweight model PHRF-RTDETR

#### 2.2.1 The base model RT-DETR

**Figure 2:**
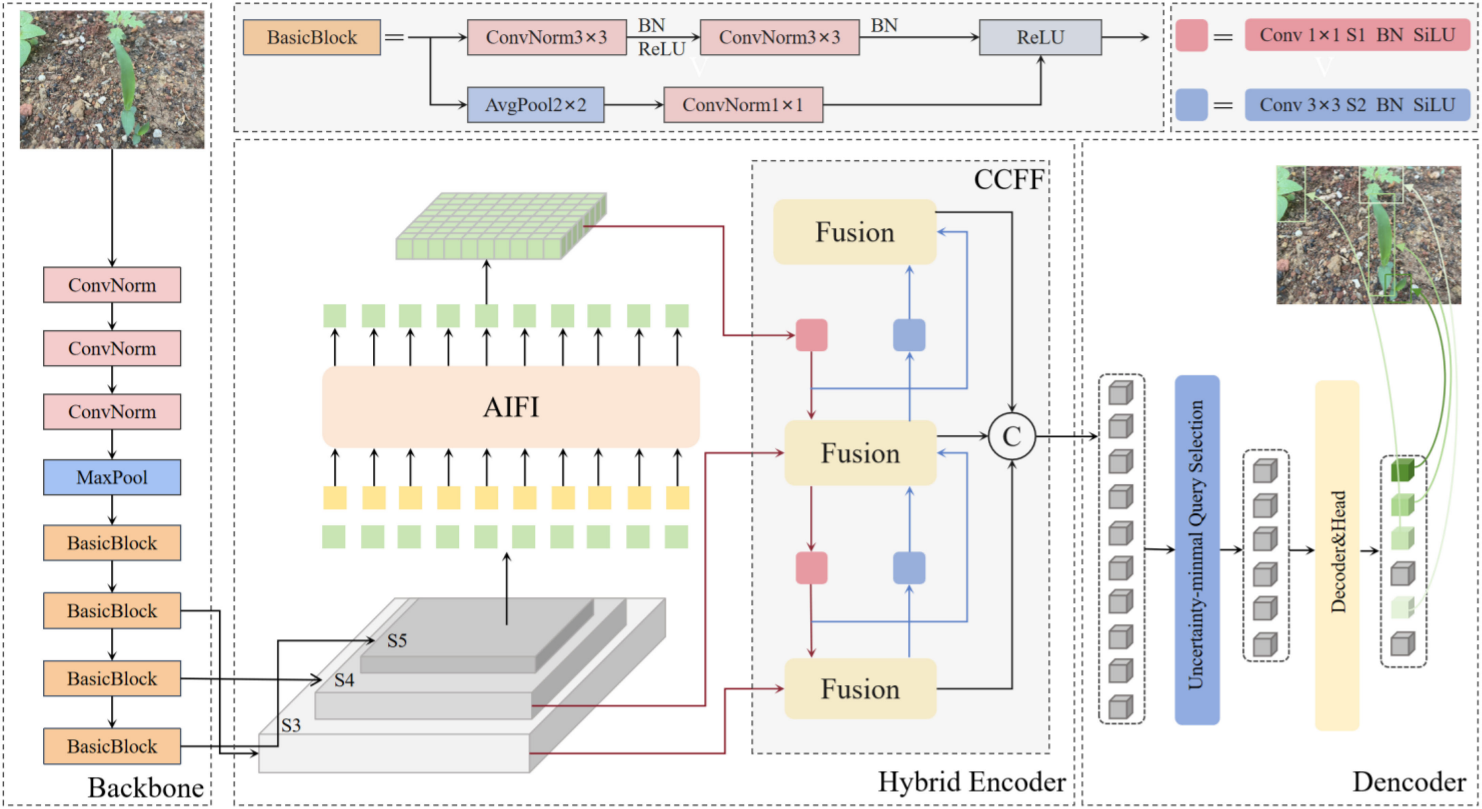
RT-DETR structure framework.

RT-DETR leverages the complementary strengths of CNN for local feature extraction and Transformer (Carion et al., 2020) for global context modeling, allowing it to effectively discern intricate relationships between weeds and their surrounding backgrounds, thus improving the detection accuracy. Additionally, RT-DETR bypasses the non-maximum suppression (NMS) step commonly employed in conventional object detection pipelines (Zhao et al., 2024), enabling the direct prediction of detection results and significantly enhancing the computational efficiency. Therefore, it is feasible to use RT-DETR as the benchmark model for weed detection in upland rice environment. As shown in **Figure 2**, RT-DETR consists of three components: a backbone network, a hybrid encoder, and a decoder.

1. Backbone: The backbone network extracts feature at different scales, providing a rich set of stage-specific feature representations for subsequent feature fusion.
2. Hybrid encoder: The encoder consists of two modules. The first is the Attention-Scale Feature Interaction (AIFI) module, based on the Transformer architecture, which performs feature interaction and refinement within scale to enhance feature representation. The second is the Cross-Scale Feature Fusion (CCFF) module, based on CNN, which effectively integrates feature information across different scales. Together these modules provide more accurate image feature inputs for the final output of the subsequent decoder.
3. Decoder: The decoder in RT-DETR is a Transformer-based denoising decoder. Initially, an object query selects a fixed number of encoder features using an uncertainty minimization mechanism. The decoder is then combined with auxiliary prediction heads to iteratively refine these queries. These queries guide the decoder in extracting valid target information from the image features provided by the encoder, ultimately generating target categories and bounding boxes.

#### 2.2.2 The PHRF-RTDETR model structure

**Figure 3:**
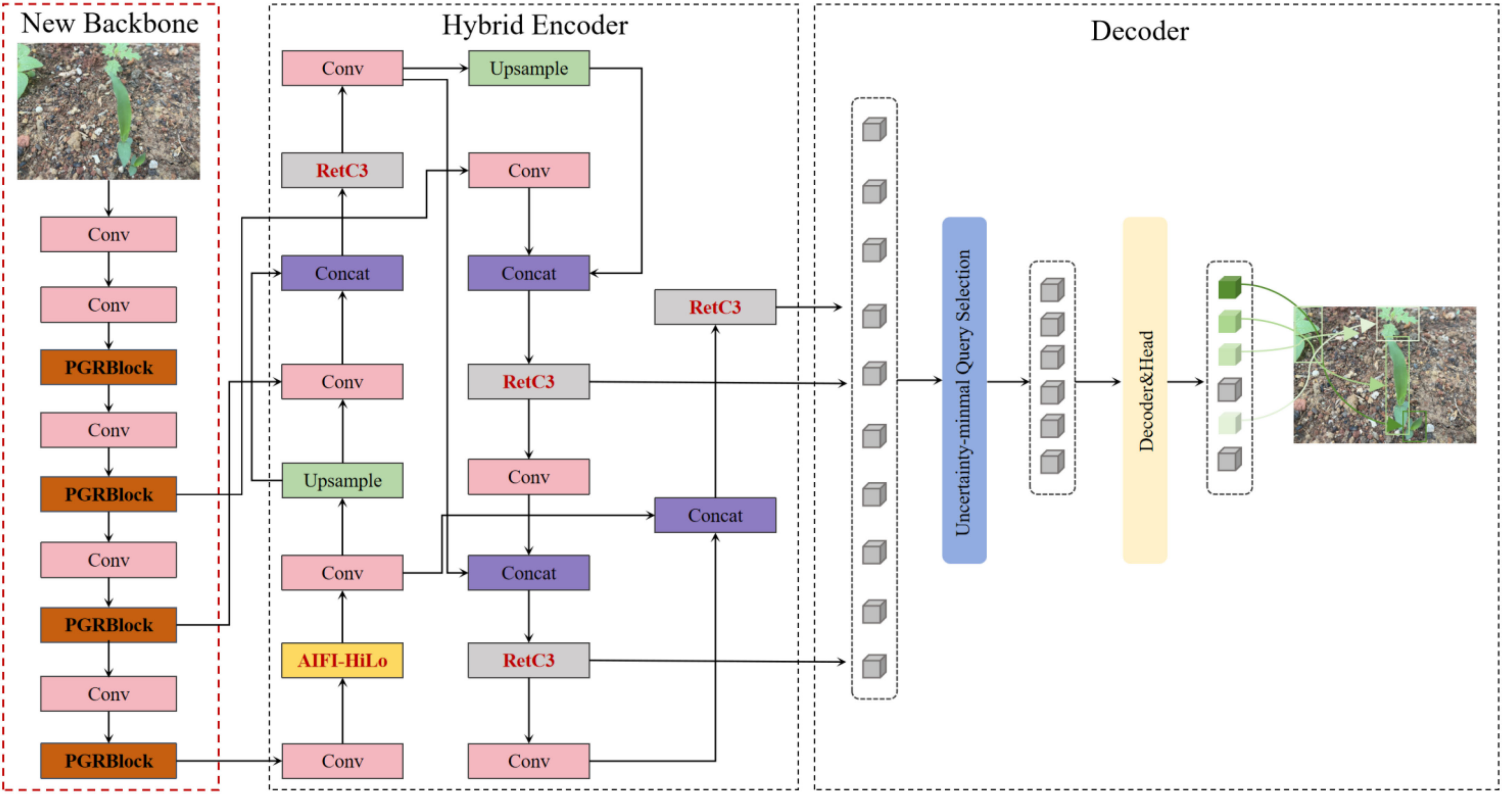
PHRF-RTDETR framework.

Although RT-DETR18, the most lightweight model in the RT-DETR series, was chosen as the base model, its backbone network architecture remains relatively complex, limiting its effectiveness in real-world weeding operations. Given that weed detection tasks in agricultural environments typically depend on hardware with limited resources and the AIFI structure, RepC3 module, and loss function in RT-DETR18 are not specifically optimized for weed detection, its practical utility in weed detection tasks of upland fields is considerably restricted. To address these challenges, we propose a more lightweight and efficient design for RT-DETR18, as illustrated in **Figure 3**.

In **Figure 3**, the new, efficient, and multi-scale lightweight backbone network, PGRNet, proposed in this study replaces the original one-layer to an eight-layer feature extraction network of RT-DETR. Next, the high- and low-frequency attentional mechanism excluding parameter growth, HiLo, is introduced into the AIFI structure, which is part of the hybrid encoder, resulting in the high-efficiency AIFI-HiLo structure. Additionally, a lightweight feature fusion structure, RetC3, is derived by combining RepC3 in CCFF with the RetBlock structure. Finally, the loss function in the detection head of the decoder is optimized by replacing the original GIoU function with the more flexible Focaler-WIoUv3 function.

#### 2.2.3 The efficient multi-scale lightweight backbone network PGRNet

The backbone network design of RT-DETR is based on the ResNet18 architecture (He et al., 2016), which has fewer layers and a more streamlined structure compared to deeper variants such as ResNet34 and ResNet50. However, ResNet18 still faces performance limitations in meeting the demands of lightweight weed detection. Therefore, we propose PGRNet, a novel lightweight backbone network centered on the PGRBlock module, designed to achieve a balance between model complexity and detection performance. By streamlining the network architecture and integrating multi-scale feature extraction capabilities, PGRNet significantly reduces computational requirements while maintaining high accuracy for weed detection in upland rice fields.

**Figure 4:**
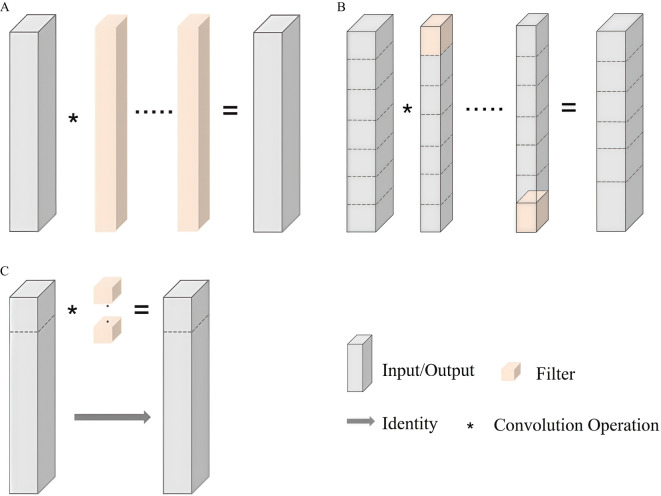
Diagrams of different convolution structures. **(A)** Standard Conv. **(B)** GConv. **(C)** PConv.

The design of PGRBlock combines the concepts of PConv (Chen et al., 2023) and GConv (Ioannou et al., 2017), both of which are lightweight techniques. The structures of GConv and PConv are illustrated in **Figures 4B, C**. In **Figure 4B**, the key idea of GConv is to divide the input data into *g* groups, with each group containing *c/g* input channels. The depth of the convolution kernel used in each group is also *c/g*, while the spatial dimensions (i.e., height and width) of the kernel remain unchanged. Each subtask performs an independent convolution operation within its respective group. The outputs from all groups are then concatenated along the channel dimension to obtain an output with *c* channels. In this manner, the number of parameters and computational cost of GConv is 1*/g* of those for standard convolution, as shown in **Figure 4A**. In **Figure 4C**, PConv selects only the first or last continuous channels in the input feature map as the representative channels for convolution operations rather than applying convolutions to all input channels. For channels not involved in the convolution, the corresponding output values directly retain the original input values. This selective convolution strategy not only optimizes memory access efficiency but also reduces computational overhead.

**Figure 5:**
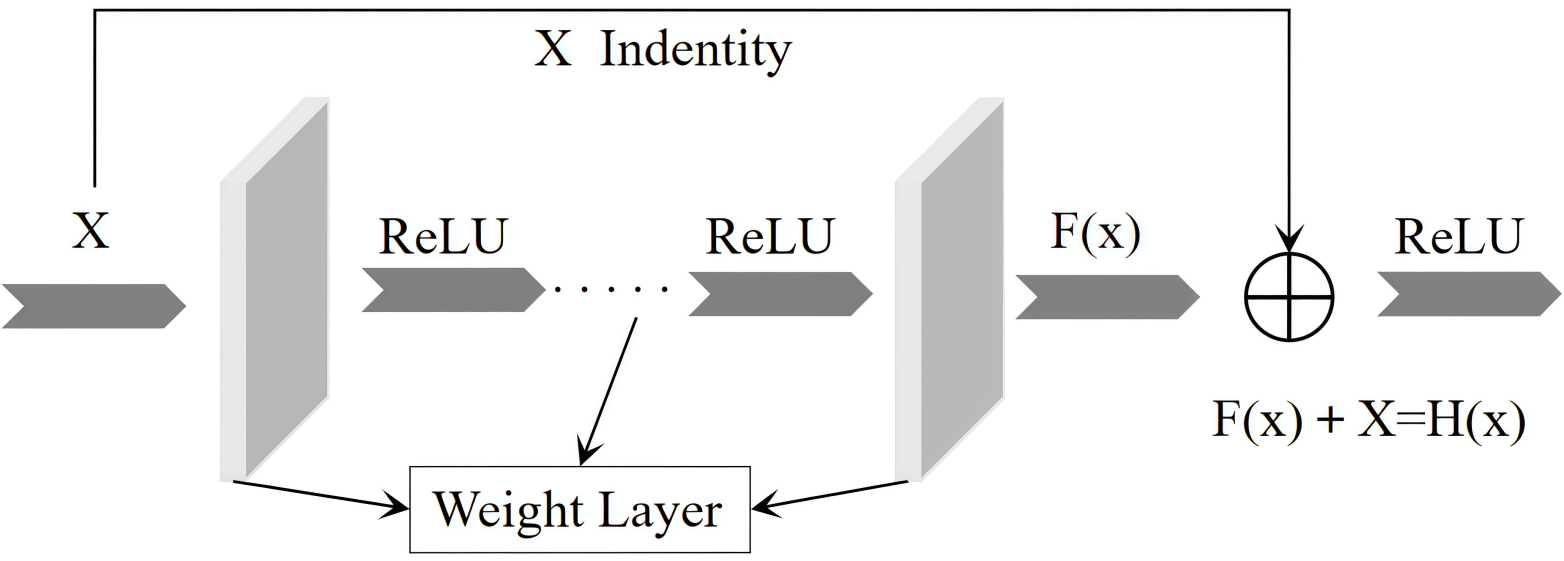
Residual structure diagram.

At the same time, the concept of residual structure is incorporated into PGRBlock to enhance the information flow in deep neural networks. The residual structure, as illustrated in **Figure 5**, is primarily composed of two components: one maintains the identity mapping, commonly referred to as the “skip connection,” which directly passes the input information *X* to the output layer. This helps prevent the gradual vanishing of information in deep networks, a phenomenon known as the vanishing gradient problem. The second component focuses on learning the residual, which represents the difference between the input *X* and the target output (*x*). By doing so, the network avoids the need to learn an entirely complex global mapping from scratch and instead only needs to correct the deviation between the input and output. This approach reduces the difficulty of model training.

**Figure 6:**
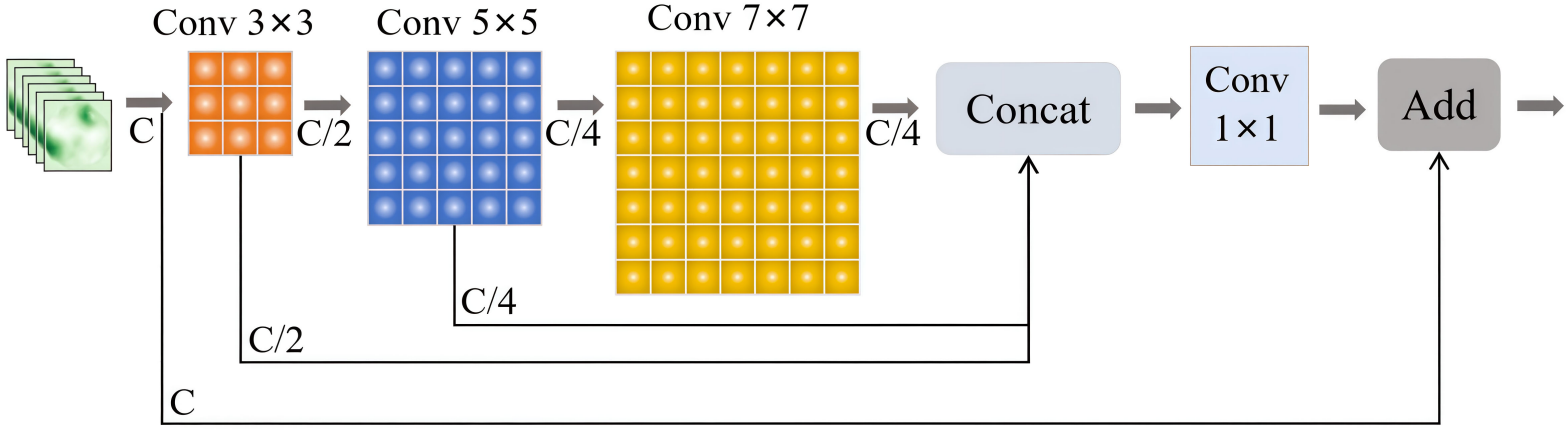
PGRBlock structure diagram.

The PGRBlock module was developed by integrating the design principles of PConv, GConv, and residual structure, resulting in an efficient and lightweight multi-scale feature extraction module. By employing convolutional kernels of different sizes, PGRBlock effectively captures multi-level features, enhancing the model’s ability to recognize hierarchical patterns. Furthermore, the module incorporates a channel grouping strategy, where convolutional operations are applied to a subset of channels while the remaining channels undergo identity mapping. This design significantly reduces computational complexity, parameter volume, and overall model size, achieving an optimized lightweight architecture. The detailed structure of PGRBlock is presented in **Figure 6**. In this figure, the feature information is first processed by a 3 × 3 convolution kernel to extract the initial feature map. Half of the channels from this feature map are then passed through a 5 × 5 convolution kernel to capture higher-level features. Next, one-quarter of the channels from the feature map processed by the 5 × 5 convolution kernel is passed into a 7 × 7 convolution kernel for a more detailed feature extraction. During the feature extraction process, feature information from different convolution kernels is concatenated. Specifically, one-quarter of the channel features from the 7 × 7 convolution kernel, one-quarter from the 5 × 5 convolution kernel, and one-half from the 3 × 3 convolution kernel are concatenated to form a complete feature map. Finally, the concatenated feature map is added to the original input feature map to produce the final output feature map.

**Figure 7:**
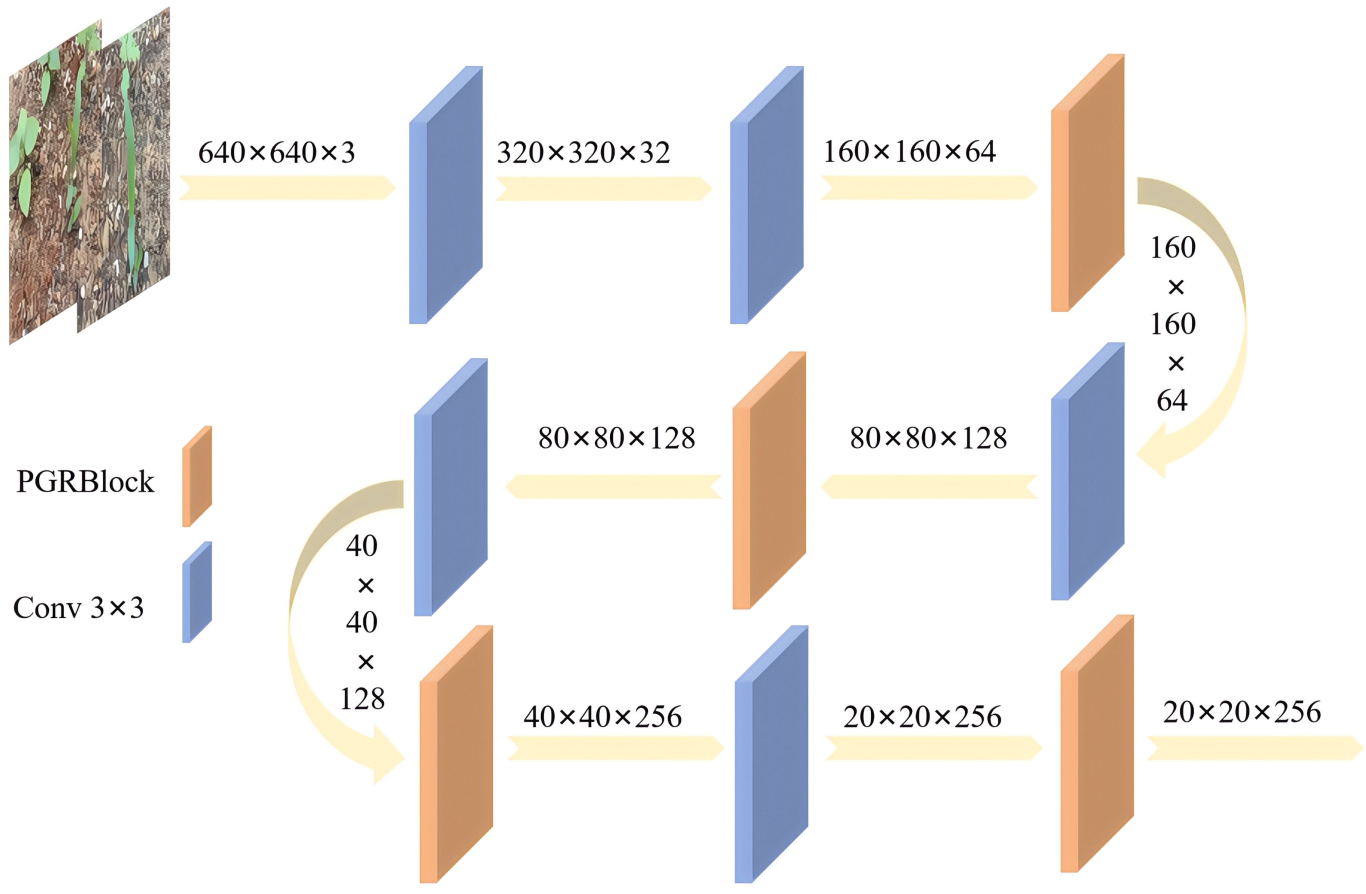
PGRNet structure diagram.

Based on PGRBlock, a new, high-efficiency, and multi-scale lightweight backbone network, PGRNet, is proposed, as shown in **Figure 7**. PGRNet consists of five 3 × 3 convolutional layers and four PGRBlock modules. In the first stage, two successive 3 × 3 convolutional layers are used to extract low-level features from the input data. PGRBlock is then applied to streamline the representation of these low-level features. In the second stage, a 3 × 3 convolutional layer is employed to extract scale-specific features from the middle layer, followed by the use of PGRBlock to eliminate redundant feature information. A final 3 × 3 convolutional layer is then applied to integrate the middle-layer features and produce the semantically enriched middle-layer output. In the third stage, PGRBlock is first used to efficiently extract high-level abstract features. A subsequent 3 × 3 convolutional layer refines these high-level semantic features, and PGRBlock is applied again to further reduce feature redundancy and refine the output.

#### 2.2.4 Improvement of attention mechanism in AIFI

**Figure 8:**
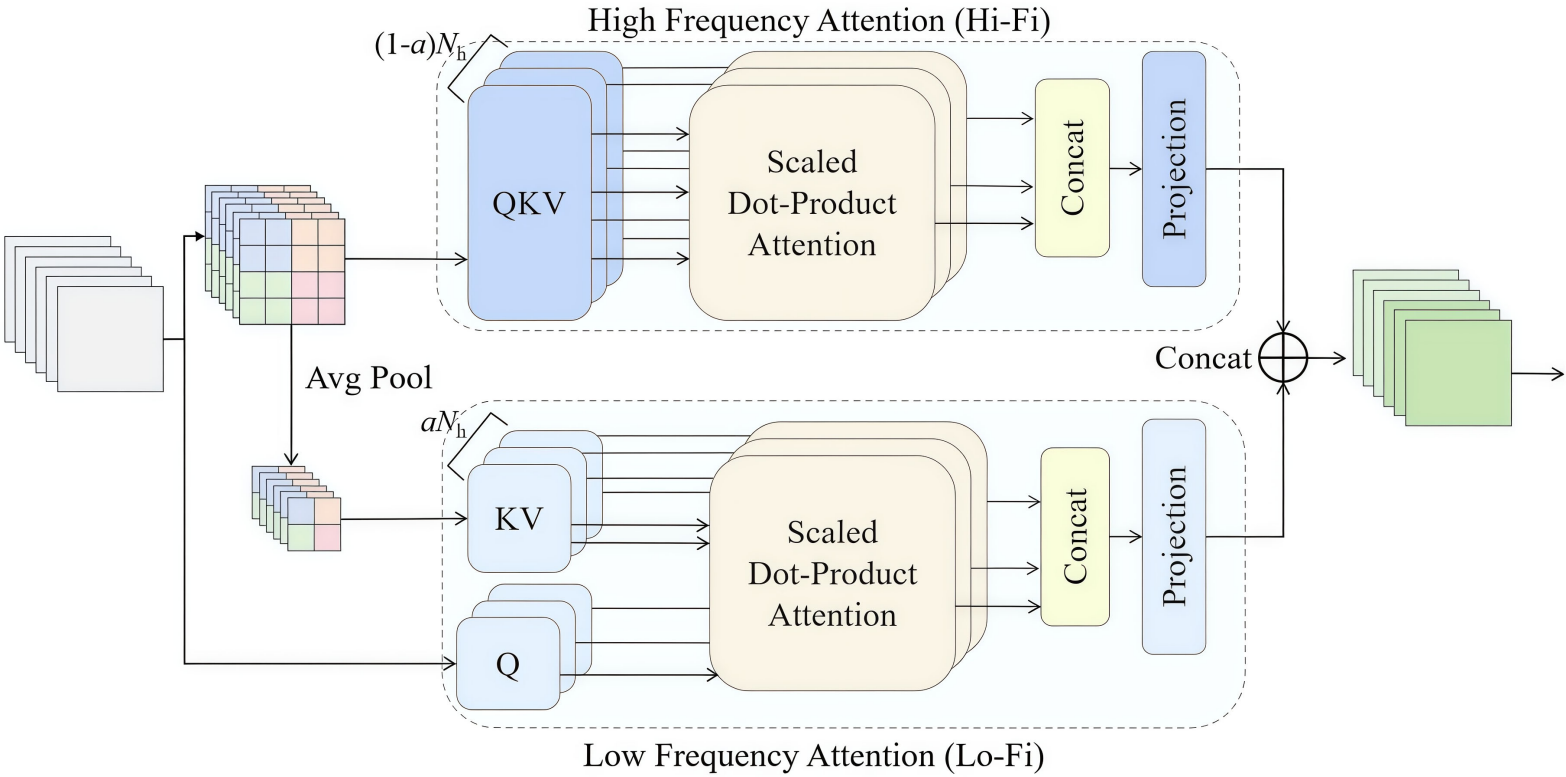
HiLo attention mechanism schematic diagram.

AIFI is an intra-scale feature interaction module based on the Transformer architecture, which captures inter-relationships between intra-scale features using the multi-head attention mechanism. However, the multi-head attention mechanism overlooks features with different frequencies, leading to the loss of high-frequency weed-related features and failing to effectively suppress the interference from low-frequency features in the upland rice background. To address this limitation, the multi-head attention mechanism was replaced with the HiLo non-parameter-added attention mechanism (Pan et al., 2022), which can focus on the local high-frequency details of weed while capturing the global low-frequency features of upland rice fields. This modification enhances the model’s performance in weed detection in upland rice. The structure of the HiLo attention mechanism is shown in **Figure 8**.

In **Figure 8**, HiLo consists of high-frequency attention (Hi-Fi) and low-frequency attention (Lo-Fi). In the Hi-Fi attention branch, the local high frequency features of weed are captured by using a simple non-overlapping local window attention mechanism in distributed (1-*a*) *N*_h_ heads. In the Lo-Fi attention branch, the average pooling operation is used to obtain the K key and V value, and then the standard attention is used to capture the global low frequency features of the upland rice field combined with the Q information of the original feature map. Finally, the weed characteristics of high- and low-frequency upland rice treated with Hi-Fi and Lo-Fi were connected, and the results were output into the CCFF structure.

#### 2.2.5 Improvement of RepC3

**Figure 9:**
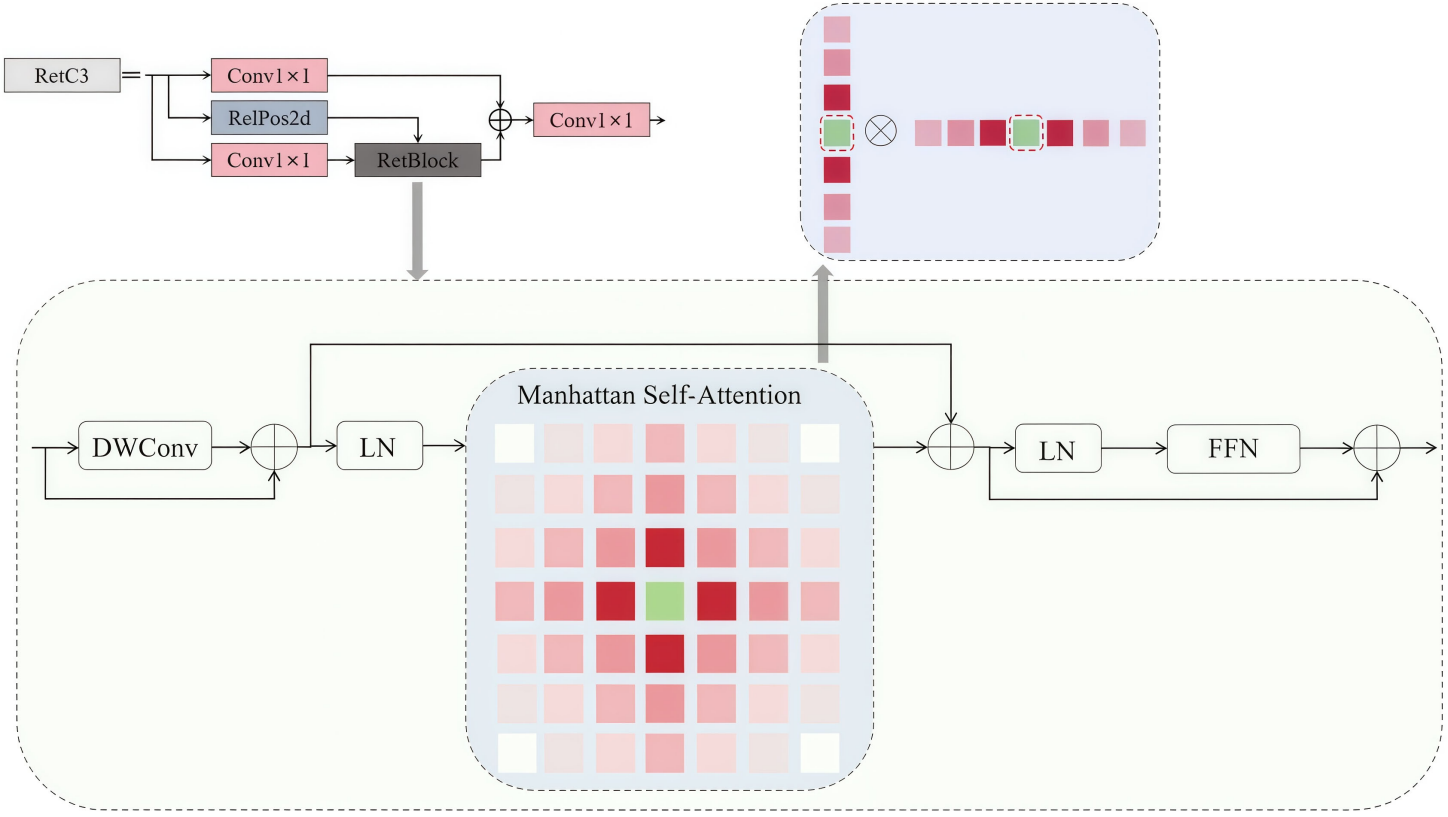
RetC3 schematic diagram.

RepC3 is a key component of the extra-scale feature fusion structure in CCFF, primarily responsible for integrating the features of upland rice weed through local convolution. However, this structure has a limitation in handling long-range dependencies. Specifically, when weeds are located in different areas of the image or are spatially isolated, the RepC3 structure struggles to capture the long-distance relationships between them, leading to reduced detection accuracy. Furthermore, the multilayer convolutional stacking in the RepC3 design increases computational and memory costs. Unlike convolutional structures based on self-attention mechanisms, which can reduce unnecessary computational redundancy through fine attention weight allocation, RepC3 lacks this capability. To address these two issues, the RetBlock (Fan et al., 2024) structure was introduced into RepC3, resulting in the RetC3 structure, which offers improved fusion performance and a lightweight design, as shown in **Figure 9**.

In **Figure 9**, the RetBlock serves as the core component of RetC3. Within the RetBlock, the input tensor x is first combined with its positional encoding and then processed by the MaSA mechanism. The MaSA utilizes a spatial attenuation matrix to model the spatial relationships between different tokens in the image, thereby enhancing the model’s ability to capture global information and long-range dependencies. Specifically, the matrix assigns attention weights based on the spatial distance between token pairs, with closer tokens receiving stronger attention and distant tokens experiencing attenuated attention. To optimize computational efficiency, MaSA decomposes the traditional two-dimensional attention mechanism into two separate one-dimensional attentions: one operating along the horizontal (row) axis and the other along the vertical (column) axis. This decomposition reduces the computational complexity from quadratic to linear, significantly lowering computational costs while maintaining the global representation of spatial features. Finally, the processed x is normalized, passed through FFN, and fused with multi-scale representations via residual connections.

#### 2.2.6 Improvement of loss function

The original IoU function used in RT-DETR is GIoU (Rezatofighi et al., 2019). However, in cases where the bounding boxes completely overlap, GIoU reduces to the traditional IoU, which limits the RT-DETR model’s ability to handle scenarios with substantial variations in weed sizes. Furthermore, it leads to inadequate boundary regression performance for occluded weeds and is prone to generating inaccurate detection results in cases of ambiguous boundaries.

To address this limitation, this study combines WIoUv3 (Tong et al., 2023) with Focaler-IoU (Zhang and Zhang, 2024) to obtain the Focaler-WIoUv3 function, which replaces the original GIoU. WIoUv3 is an improvement over WIoUv, retaining its advantage of reducing the negative impact of low-quality samples on the training process by minimizing the penalty from geometric mismatches. Additionally, WIoUv3 introduces a non-monotonic focusing coefficient *r*, which allows the model to focus more effectively on difficult samples while suppressing the adverse gradients caused by low-quality anchor frames. Based on WIoUv3, the IoU*^focaler^* is integrated to develop Focaler-WIoUv3. This advanced function significantly enhances bounding box regression accuracy, providing robust handling of weed samples characterized by size variations, occlusions, and indistinct boundaries. The Focaler-WIoUv3 formula is shown in Equations 1–4.


(1) $$L_{Focaler-WIoUv3}=L_{WIoUv3}+IoU-IoU^{Focaler}$$

(2) $$L_{WIoUv3}=rL_{WIoUv1}$$

(3) $$L_{WIoUv1}=R_{WIoU}L_{IoU}$$

(4) $$IoU^{Focaler}=\{\begin{array}{c} 0,IoU\ll d\\ \frac{IoU-d}{u-d},\\ 1,IoU>d \end{array}d\ll IoU\ll u$$

### 2.3 Experimental platform

The software environment for this experiment includes the PyTorch 2.4.1 deep learning framework, with programming language conducted in Python 3.8.19. Development tool was carried out using PyCharm Community Edition, and the system is accelerated by CUDA 11.8. The hardware configuration consists of an NVIDIA GeForce RTX 4090 graphics card and an Intel(R) Xeon(R) Silver 4214R CPU (2.40 Hz).

For the experimental setup, a total of 300 iterations were performed, with a training batch size of eight. The initial learning rate was set to 0.0001.

### 2.4 Evaluation metrics

To comprehensively evaluate the performance of the PHRF-RTDETR weed detection model in upland rice, the selection of evaluation metrics focused primarily on model lightweight and accuracy. Accuracy-related metrics include precision (P), recall (R), mean average precision (mAP), and F1 score. The corresponding formulas are provided in Equations 5–9.


(5) $$Precision=\frac{TP}{TP+FP}$$

(6) $$Recall=\frac{TP}{TP+FN}$$

(7) $$AP=\int_{0}^{1}P\left(R\right)dR$$

(8) $$mAP=\frac{1}{n}\sum_{i=1}^{n}AP_{i}$$

(9) $$F_{1}=\frac{2\times P\times R}{P+R}$$

where *TP* refers to the number of weed correctly identified by the model, *TN* refers to the number of non-weed (e.g., other crops, backgrounds, etc.) correctly identified as non-weeds, *FP* refers to the number of non-weed incorrectly identified as weed, and *FN* refers to the number of weed incorrectly classified as non-weed. The closer the values of P, R, mAP, and F1 score are to 1, the better the accuracy in weed detection tasks.

The lightweight-related metrics include number of parameters (Params), floating-point operations (FLOPs), and model size (MB). The corresponding formulas are provided in Equations 10–12:


(10) $$Params=K_{w}\times K_{h}\times C_{out}\times C_{in}$$

(11) FLOPs=H×W×Params

(12) $$Weight\;Size=\frac{Params\times B_{Params}}{1024^{2}}$$

where *C _out_* and *C_in_* denote the number of output and input channels of the convolutional kernel, *K_w_* and *K_h_* represent the width and height of the convolutional kernel, *H _out_* and *W_out_* refer to the height and width of the output feature map, respectively, and *B_params_* represents the number of bytes occupied by each weight. Smaller values of FLOPs, Params, and MB indicate better performance in terms of computational efficiency and storage requirements.

## 3 Results and analysis

### 3.1 The comparison experiment of lightweight backbone

To evaluate the effectiveness of PGRNet proposed in this study, we conducted comparative experiments with several representative lightweight backbone networks. These include RepViT (Wang A. et al., 2024) and EfficientViT (Liu et al., 2023), which are based on the Vision Transformer (ViT), as well as UniRepLKNet (Ding et al., 2024) and LSKNet (Li et al., 2023), which are based on large kernel convolutions. Additionally, we compared PGRNet with lightweight backbone networks based on CNN, such as StarNet (Ma et al., 2024), MobileNetV4 (Qin et al., 2025), ConvNeXtV2 (Woo et al., 2023), and Fasternet. By performing a comprehensive comparison with these various lightweight backbone networks, we thoroughly assessed the advantages of PGRNet in terms of lightweight, as shown in **Table 2**.

**Table 2:** Comparative performance of various lightweight backbone networks.

Backbone	P/%	R/%	mAP50/%	mAP50:95/%	FLOPs/G	Params/M	Size/MB
Basic	91.5	86.3	88.2	78.1	57	19.88	38.6
RepViT	92.3	83.2	86.9	75.8	36.3	13.31	26.4
EfficientViT	87.6	82.9	85.1	74.4	27.2	10.71	21.6
UniRepLKNet	91.8	85.3	89.6	78.5	33.4	12.71	25.5
LSKNet	89.7	85.9	87.1	76.1	37.6	12.57	24.7
StarNet	92.6	86.5	88.8	77.4	29.7	11.22	22.1
MobileNetV4	91	83.8	87.8	76.2	39.5	11.32	22.3
ConvNeXtV2	89	82.7	87.5	75.7	31.9	12.31	24.1
Fasternet	91.9	87.3	88.6	77.6	37.2	14.34	28
PGRNet	89.7	83.9	87.7	77	29.9	10.58	20.8

As shown in **Table 2**, compared to the original backbone network of RT-DETR, all of the lightweight backbone networks exhibit varying degrees of reduction in FLOPs, Params, and Size, thereby achieving a lightweight effect. Among the ViT-based lightweight networks, EfficientViT demonstrates the best lightweight performance, with reductions of 9.1 G in FLOPs, 2.6 M in Params, and 4.8 MB in Size compared to RepViT. In comparison to EfficientViT, the lightweight effect of PGRNet is almost identical, while its weed detection accuracy metrics of P, R, mAP50, and mAP50:95 increased by 2.1 percentage points, 1 percentage point, 2.6 percentage points, and 2.6 percentage points, respectively. When compared to lightweight networks based on large kernel convolutions, PGRNet exhibits the best lightweight performance, with detection accuracy in upland rice comparable to LSKNet. Among CNN-based lightweight networks, StarNet achieves both the best detection accuracy for weed and the most efficient lightweight performance. However, PGRNet outperforms StarNet in terms of parameter count and model size, with reductions of 0.64 M and 1.3 MB, respectively.

In summary, PGRNet, an efficient multi-scale lightweight backbone network that integrates the advantages of PConv, GConv, and residual connections, demonstrates superior overall performance compared to existing mainstream lightweight backbone networks. In terms of weed detection accuracy, PGRNet achieves a reasonable level, while its lightweight indices are outstanding. Specifically, PGRNet’s FLOPs, Params, and Size are 29.9 G, 10.58 M, and 20.8 MB, respectively.

### 3.2 The validation experiment of AIFI-HiLo

**Figure 10:**
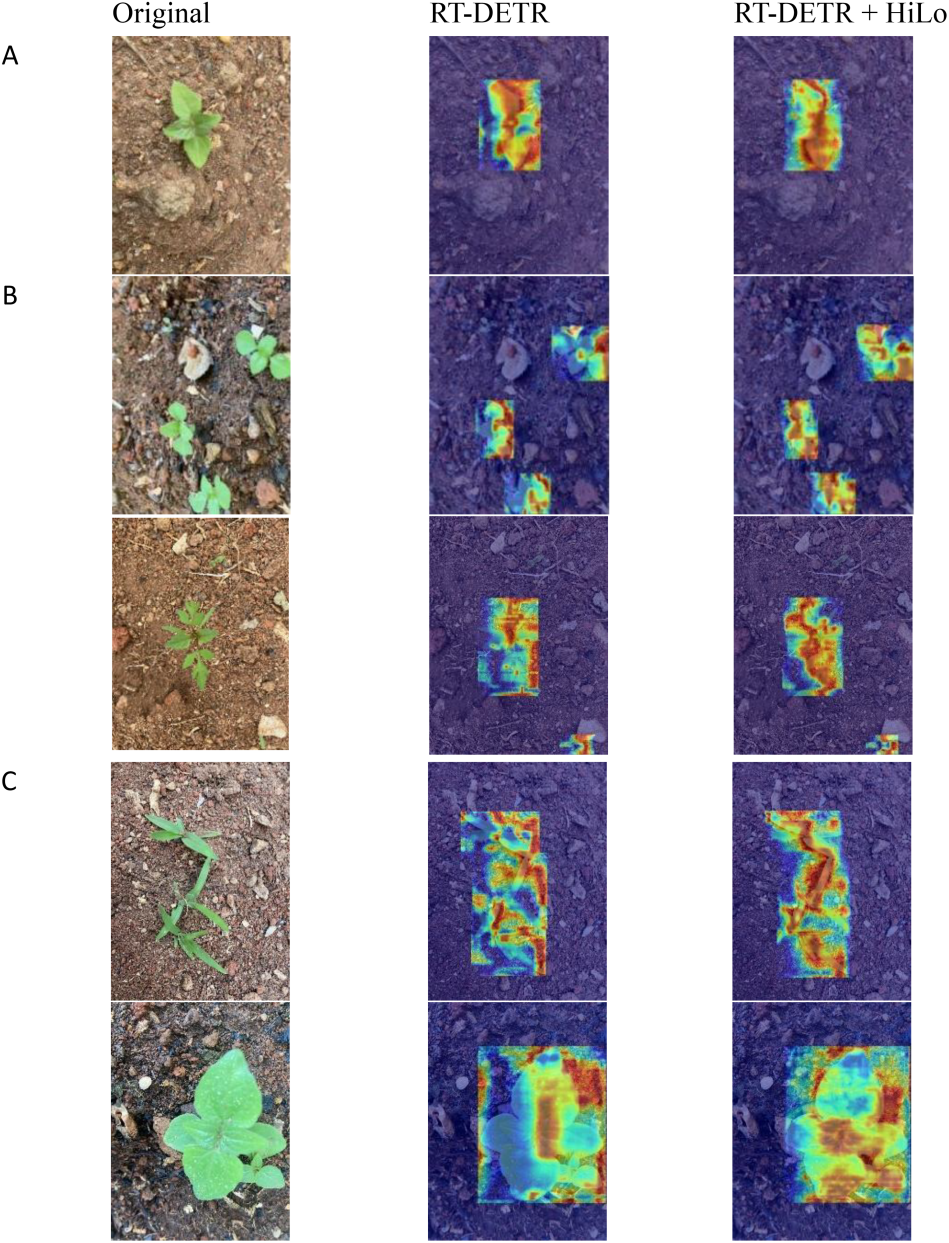
Comparison of thermal maps after adding HiLo. **(A)** Single target. **(B)** Multiple target. **(C)** Occlusion.

The decision-making process of a deep learning model is often referred to as a “black box”, which means that the internal reasoning mechanism is difficult to directly interpret. By visualizing the model’s attention distribution, the Grad-CAM++ (Chattopadhyay et al., 2018) heatmap experiment can intuitively illustrate how the attention distribution was adjusted in the weed detection task for upland rice after incorporating HiLo. This adjustment enables the model to focus on the more meaningful features of weed. The heatmap is shown in **Figure 10**.

As shown in **Figure 10**, although the basic model RT-DETR is able to focus on weed areas, it still exhibits some misfocused regions. Specifically, the RT-DETR model allocates unnecessary attention to non-target areas, such as the background of upland rice fields or the gaps between weed. After incorporating the HiLo attention mechanism, the attention distribution of the RT-DETR model improved significantly. The attention area for weed targets increased markedly, while the attention to non-target areas was substantially reduced. This improvement is due to the HiLo mechanism, in which the upper branch (Hi-Fi) captures fine-grained, high-frequency weed features through a local window self-attention mechanism, while the lower branch (Lo-Fi) captures the low-frequency global features of the dryland field. Notably, in complex scenarios characterized by multiple targets and occlusions, the HiLo-enhanced model demonstrates the capability to dynamically allocate attention, enabling it to accurately capture the features of multiple weeds and effectively address occluded areas.

In summary, the HiLo mechanism enables the RT-DETR model to effectively focus on weed features at different frequencies, thereby enhancing the accuracy of weed detection task in upland rice.

### 3.3 The validation experiment of RetC3 lightweight performance

To verify whether RetC3 addresses the issues of insufficient lightweight performance and detection accuracy caused by RepC3’s redundancy in feature calculation and its inability to effectively capture long-range relationships in weed, we combined DiverseBranchBlock (Ding et al., 2021), Conv3XC (Wan et al., 2024), and gConvBlock (Song et al., 2022) with RepC3 to form DBBC3, Conv3XCC3, gConvC3, and RetC3 for comparison experiments, as shown in **Table 3**.

**Table 3:** Performance of different RepC3 types.

Type	mAP50/%	mAP50:95/%	FLOPs/G	Params/M	Size/MB
Basic	88.2	78.1	57	19.88	38.6
DBBC3	89	78.8	49.1	18.31	38.3
Conv3XCC3	88.8	78.2	57	19.88	39.3
gConvC3	88.2	77.8	57.1	18.72	35.9
RetC3	89.8	79	50.2	18.52	35.6

As shown in **Table 3**, compared to the original RepC3 structure, Conv3XCC3 showed minimal improvement in lightweight performance, and the accuracy of weed detection in upland rice was not significantly enhanced. In contrast, while the FLOPs of gConvC3 increased by 0.1 G, the Params decreased by 5.8%, and the Size was reduced by 7%; the accuracy of weed detection slightly declined. Compared to the original RepC3 structure, Conv3XCC3 and gConvC3, DBBC3 achieved the best accuracy metrics for weed detection, with mAP50 and mAP50:95 values of 89% and 78.8%, respectively. Additionally, the FLOPs, Params, and Size of DBBC3 were reduced by 7.98 G, 1.57 M, and 0.3 MB, respectively, compared to the original RepC3 structure. When compared to DBBC3, RetC3 showed improvements in accuracy, with mAP50 and mAP50:95 increasing by 0.8 and 0.2 percentage points, respectively, while retaining similar lightweight performance metrics.

In conclusion, compared to other improved RepC3 structures, RetC3 achieves the best weed detection accuracy and lightweight performance for upland rice. Compared to the original RepC3, RetC3 demonstrates improvements with mAP50 and mAP50:95 values of 89.8% and 79%, respectively, representing increases of 1.6 and 0.9 percentage points. Additionally, the FLOPs, Params, and Size are reduced by 11.9%, 6.8%, and 7.8%, respectively. Thus, RetC3 successfully meets the objectives of reducing computational redundancy while enhancing weed detection accuracy.

### 3.4 The comparison experiment of loss function

**Figure 11:**
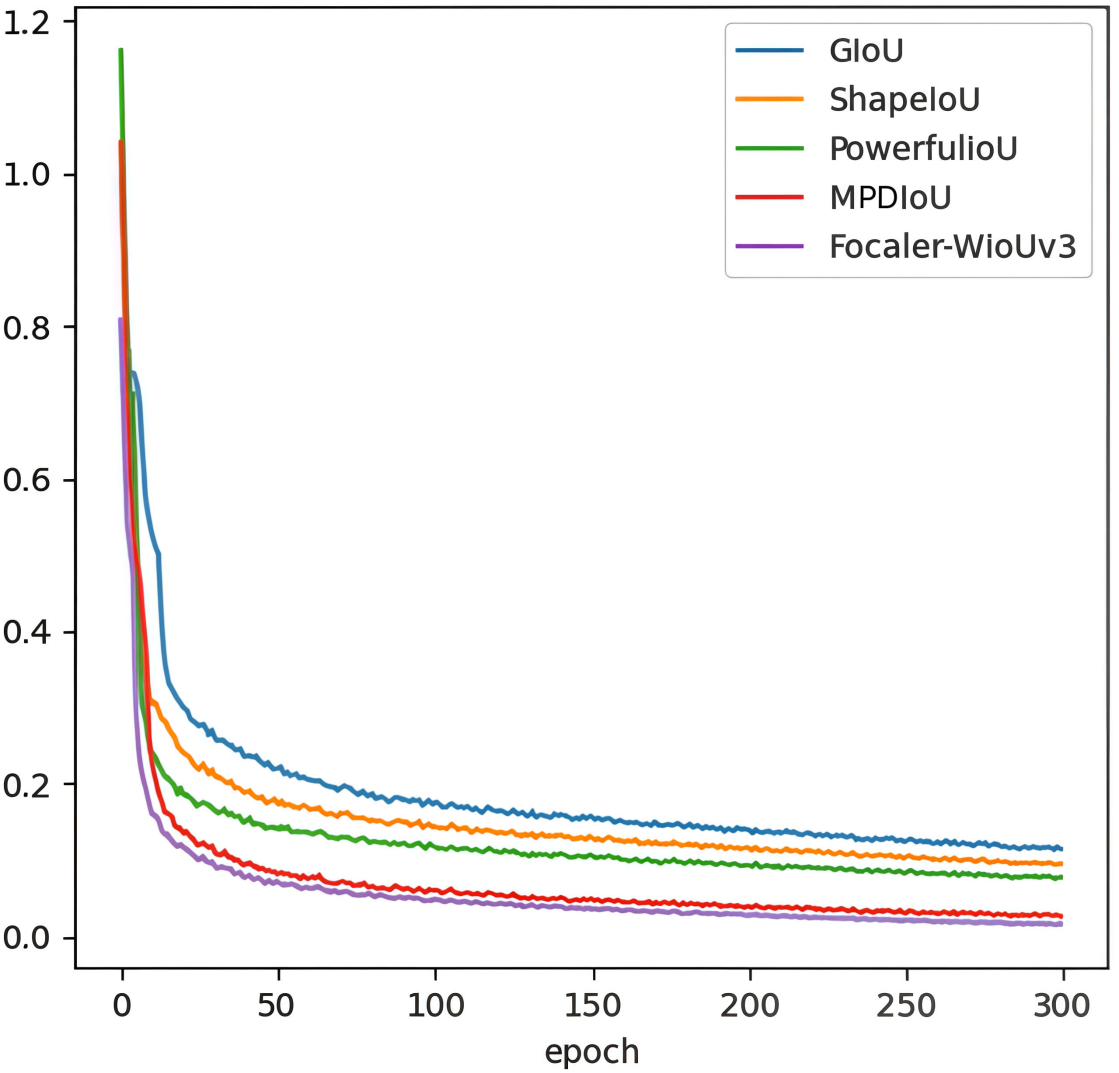
Comparison of the curves for different loss functions.

To verify whether the Focaler-WIoUv3 function can dynamically adjust the border regression for different samples, optimize the training process of the RT-DETR model, and thereby improve the generalization ability in detecting weeds in upland rice, a comparative experiment was conducted using GIoU, ShapeIoU (Zhang and Zhang, 2023), PowerfulIoU (Liu et al., 2024), and MPDIoU (Ma and Xu, 2023). The results of this experiment are presented in **Table 4** and **Figure 11**.

**Table 4:** Performance of different loss functions.

Loss function	P/%	R/%	mAP50/%	mAP50:95/%
GIoU	91.5	86.3	88.2	78.1
ShapeIoU	92.2	84.5	89.2	78.8
PowerfulIoU	92	85.1	88.5	77.9
MPDIoU	92.7	85.3	89.7	78.9
Focler-WIoUv3	92.6	87.2	89.9	79.3

As shown in **Table 4**, compared to the original loss function GIoU, the accuracy metrics P, R, mAP50, and mAP50:95 for Focalcer-WIoUv3 in weed detection for upland rice increased by 1.1, 0.9, 1.7, and 1.2 percentage points, respectively. Among the other loss functions, MDPIoU outperformed ShapeIoU and PowerfulIoU, achieving the highest values for P, R, mAP50, and mAP50:95 at 92.7%, 85.3%, 89.7%, and 78.9%, respectively. When compared to MDPIoU, Focaler-WIoUv3 showed a similar performance for P but exhibited increases of 1.9 percentage points in R, 0.2 percentage points in mAP50, and 0.4 percentage points in mAP50:95.

As shown in **Figure 11**, all loss functions exhibit relatively strong oscillations in the early stages. However, as training progresses, the oscillations gradually decrease. Among them, Focaler-WIoUv3 exhibits the earliest disappearance of oscillation, indicating that its convergence rate is the fastest. Additionally, its stability is reflected in the smoothness of the curve. Compared to GIoU, ShapeIoU, PowerfulIoU, and MDPIoU, Focaler-WIoUv3 demonstrates the smoothest loss curve, suggesting that the training process of the RT-DETR model, guided by Focaler-WIoUv3, is the most stable.

In conclusion, the Focaler-WIoUv3 function enables the RT-DETR model to demonstrate superior generalization ability in the weed detection task for upland rice.

### 3.5 The ablation experiment of overall improvement

**Figure 12:**
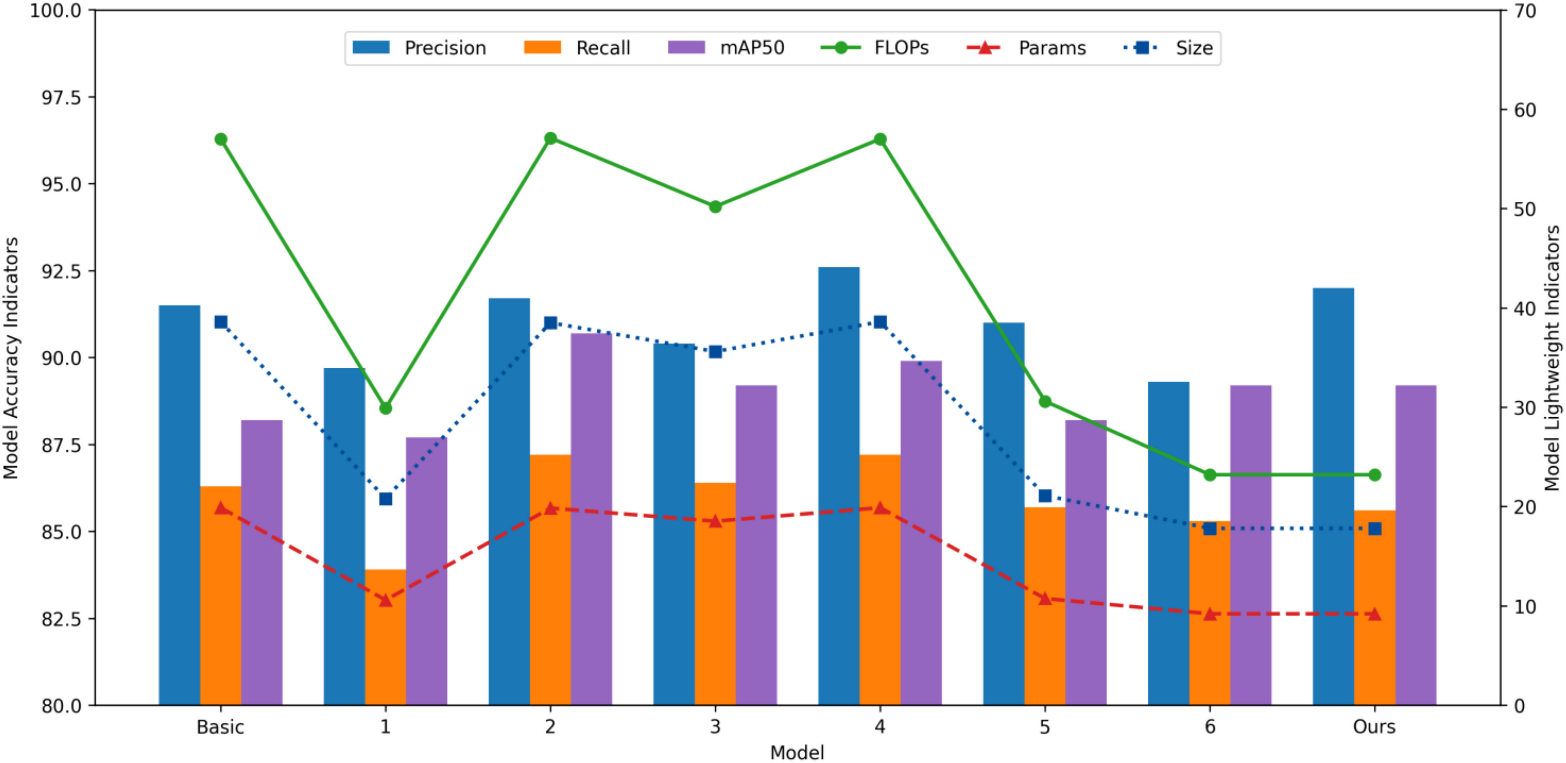
Comprehensive comparison of different improvement points.

To assess the impact of each improved module on the lightweight and accuracy performance of the RT-DETR model for weed detection in the upland rice, an ablation experiment was conducted. Models 1 through 4 correspond to the ablation experiments of individual improved modules, while models 5 and 6 combine the first two and three modules, respectively. Finally, the PHRF-RTDETR model incorporates all four improved modules. The results are presented in **Table 5** and **Figure 12**.

**Table 5:** Performance of different improved points.

Model	PGRNet	AIFI-HiLo	RetC3	Focaler-WIoUv3	P/%	R/%	mAP50/%	FLOPs/G	Params/M	Size/MB
Basic	–	–	–	–	91.5	86.3	88.2	57	19.88	38.6
1	✓	–	–	–	89.7	83.9	87.7	29.9	10.58	20.8
2	–	✓	–	–	91.7	87.2	90.7	57.1	19.84	38.5
3	–	–	✓	–	90.4	86.4	89.8	50.2	18.52	35.6
4	–	–	–	✓	92.6	87.2	89.9	57	19.88	38.6
5	✓	✓	–	–	91	85.7	88.2	30.6	10.74	21.1
6	✓	✓	✓	–	89.3	85.3	89.2	23.2	9.2	17.8
Ours	✓	✓	✓	✓	92	85.6	88.2	23.2	9.2	17.8

As shown in **Table 5**, compared with the baseline model, the P and R indices of model 1, after incorporating PGRNet, decreased slightly. However, the FLOPs, Params, and Size were reduced by 47.5%, 46.7%, and 46.1%, respectively. This demonstrates that PGRNet significantly simplifies the model through its lightweight convolution and residual connections, with only a minimal decrease in accuracy. According to **Figure 12**, model 3, which incorporates RetC3, exhibits a similar trend to the previous model, with a slight decrease in the lightweight indices. This is due to the RetBlock in RetC3, which reduces computational complexity to linear levels through decomposition of the self-attention mechanism and assigns different attention weights based on the spatial distance between token pairs to ensure detection accuracy. Both model 2, which incorporates AIFI-HiLo, and model 4, which incorporates Focaler-WIoUv3, achieved optimization of the weed detection accuracy indexes in upland rice without increasing the lightweight indexes. This is because HiLo is a no-parameter-added attention mechanism, and optimizing the loss function typically does not incur additional computational overhead.

As presented in **Table 5**, the incorporation of RetC3 into model 5 resulted in the development of model 6. By incorporating PGRNet, AIFI-HiLo, and RetC3, model 6 achieved optimal lightweight performance, with FLOPs, Params, and model size reduced by 59.3%, 53.7%, and 53.9%, respectively, compared to the baseline. With the addition of the Focaler-WIoUv3 loss function, the final model maintained a mAP50 of 88.2, unchanged from the baseline, while precision increased by 0.5 percentage points and recall decreased by 0.7 percentage points. Compared to the baseline model, the accuracy metrics remained close to their original levels.

To sum up, the weed detection accuracy of the PHRF-RTDETR model for upland rice remained largely unchanged, with P, R, and mAP50 values of 92%, 85.6%, and 88.2%, respectively. The lightweight indices, including FLOPs, Params, and Size, were significantly reduced to 23.2 G, 9.2 M, and 17.8 MB, respectively. This demonstrates a successful balance between model detection accuracy and lightweight efficiency.

### 3.6 Comprehensive evaluation of the classification performance of PHRF-RTDETR

**Figure 13:**
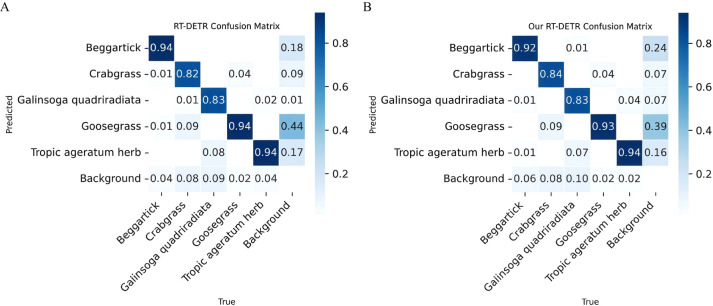
Confusion matrix contrast diagram. **(A)** RT-DETR confusion matrix. **(B)** PHRF-RTDETR confusion matrix.

**Figure 14:**
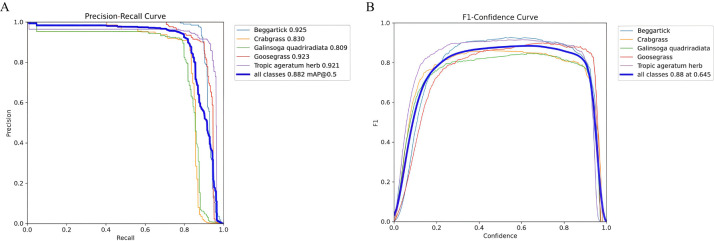
Visual comparison of mAP50 and F1 indicators. **(A)** mAP50 visual figure. **(B)** F1 visual figure.

To evaluate the classification performance of the PHRF-RTDETR model on five types of weed in upland rice, a comprehensive analysis was conducted using tools such as the confusion matrix, etc. The results are presented in **Figures 13** and **14** and **Table 6**.

**Table 6:** Performance of different species of weed.

Class	P/%	R/%	mAP50/%	mAP50:95/%
All	92	85.6	88.2	76.6
Beggartick	98.5	87	92.5	77.5
Crabgrass	90.7	80	83	73.1
*Galinsoga quadriradiata*	91.1	79.3	80.9	71
Goosegrass	88	90.1	92.3	82.1
Tropic ageratum herb	91.5	91.4	92.1	79.5

The depth of the color blocks in the confusion matrix represents various classification outcomes, with darker colors indicating a higher frequency of corresponding classification results. As shown in **Figures 13A, B**, the confusion matrices of both the original model and the PHRF-RTDETR model indicate that the detection accuracy for beggartick, goosegrass, and tropic ageratum herb is above 0.92, while the accuracy for crabgrass and *Galinsoga quadriradiata* is above 0.82. All off-diagonal values represent misclassification detection. The confusion matrices of the original and PHRF-RTDETR models reveal minimal differences in the classification of the five weed species. Overall, the classification performance of the PHRF-RTDETR model, achieved through lightweight improvements to the foundational RT-DETR model, remains stable without decline in overall efficacy.

In **Figure 14A**, the area enclosed by the P/R curve represents the detection performance of the PHRF-RTDETR model on different weed species under IoU of 0.5. The species are ranked from highest to lowest detection performance as follows: beggartick, goosegrass, tropic ageratum herb, *Galinsoga quadriradiata*, and crabgrass. In **Figure 14B**, the F1 score, which is the harmonic mean of P and R, reaches a value of 0.88 for all weeds in upland rice, under a confidence level of 0.645.

In **Table 6**, the PHRF-RTDETR model achieved an overall P of 92% for all types of weeds. Among these, the P for detecting beggartick was the highest at 98.5%, demonstrating that PHRF-RTDETR is particularly effective at detecting this species. The R for all weed species was 85.6%, with the lowest R at 79.3%, indicating that the PHRF-RTDETR missed most instances of *Galinsoga quadriradiata*. The mAP50 and mAP50:95 for detecting the five types of weeds were 88.2% and 76.6%, respectively, reflecting that PHRF-RTDETR successfully and efficiently detected the five weed categories when the predicted bounding box overlapped with the ground truth by more than 50% or between 50% and 95%.

In conclusion, the PHRF-RTDETR model demonstrates robust performance in classifying five types of weeds in upland rice, further confirming its superiority.

### 3.7 Comparative analysis of different series models

**Figure 15:**
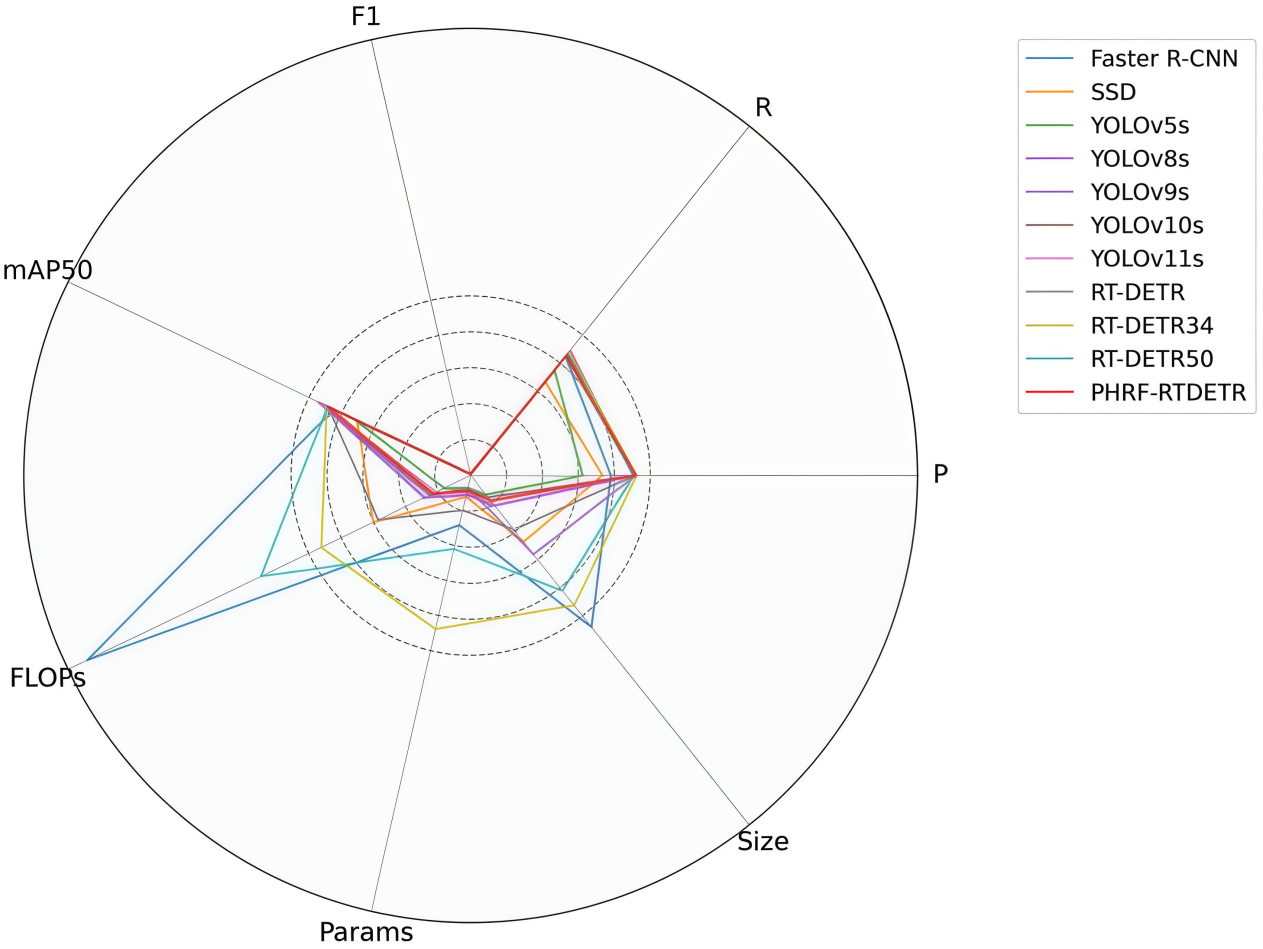
Visual comparison of the different models.

To further validate the superiority of the PHRF-RTDETR model in detecting weed in upland rice, several other advanced target detection models were selected for comparative experiments. These included traditional models such as Faster R-CNN and SSD, YOLO series including YOLOv5s, YOLOv8s, YOLOv9s, YOLOv10s, and YOLOv11s, and the RT-DETR series such as RT-DETR, RT-DETR34, and RT-DETR50, as shown in **Table 7** and **Figure 15**.

**Table 7:** Performance of different series models.

Model	P/%	R/%	mAP50/%	F1	FLOPs/G	Params/M	Size/MB
Faster R-CNN	78.1	84.8	84.6	0.81	237.04	28.32	108
SSD	73.5	66.7	70.2	0.7	60.12	12.2	47.2
YOLOv5s	62.4	75	71.6	0.66	16.3	7.06	13.7
YOLOv8s	91.7	86.6	93.4	0.89	28.4	11.13	21.4
YOLOv9s	91.8	88	94.3	0.9	26.2	7.07	56.2
YOLOv10s	91.3	84.9	92.2	0.88	24.5	8.04	15.7
YOLOv11s	90.2	89.3	93.5	0.9	21.3	9.41	18.3
RT-DETR	91.5	86.3	88.2	0.89	57	19.88	38.6
RT-DETR34	92.4	87.8	89.1	0.9	92.4	87.8	92.4
RT-DETR50	90.9	86.3	88.4	0.88	129.6	41.96	82.1
PHRF-RTDETR	92	85.6	88.2	0.88	23.2	9.2	17.8

As shown in **Table 7**, in the traditional target detection models, Faster R-CNN has significantly higher FLOPs, Params, and Size compared to the SSD model. Although Faster R-CNN achieves better detection accuracy for upland rice weed than SSD, it clearly does not optimize the use of computational resources. Among the YOLO series, YOLOv5s exhibits the best lightweight indices, with reductions of 42.6% and 36.6%, respectively, compared to YOLOv8s, which has the highest number of FLOPs and Params. When compared to YOLOv9s, which has the largest Size, YOLOv5s shows 75.6% reduction of size. However, YOLOv5s exhibited the lowest performance metrics, with mAP50 and F1 scores of 71.6 and 0.66, respectively. Among the RT-DETR series, RT-DETR34 demonstrated superior accuracy in weed detection in upland rice fields, achieving P, R, mAP50, and F1 scores of 92.4%, 87.8%, 89.1%, and 0.9, respectively. In comparison to RT-DETR34, RT-DETR50 required more computational resources and showed a decline in all accuracy metrics, with mAP50 decreasing by 0.7 percentage points and the F1 score dropping by 0.02.

As shown in **Figure 15**, compared to the SSD model, which exhibits the best performance among traditional target detection models, the PHRF-RTDETR model outperforms SSD in seven key dimensions FLOPs, Params, Size, P, R, mAP50, and F1. While the PHRF-RTDETR model is not as simplified as YOLOv5s, the lightest model in the YOLO series, it surpasses YOLOv5s in terms of P, R, mAP50, and F1 by 47.4%, 14.1%, 23.2%, and 33.3%, respectively. Compared with the YOLO series’ top-performing models, YOLOv10s and YOLOv11s, the PHRF-RTDETR model demonstrates a higher P value than both, and its *R* value is 0.7 percentage points higher than that of YOLOv10s. Although its mAP50 is not at an advantageous level, when considering the lightweight metrics, its FLOPs is 5.3% lower than that of YOLOv10s, and its Params and Size are 2.2% and 2.7% lower than those of YOLOv11s, respectively. Additionally, PHRF-RTDETR does not require NMS, simplifying the post-processing procedure and facilitating hardware acceleration. In conclusion, the PHRF RTDETR model offers the best overall performance, even though it may not achieve the absolute optimum in some dimensions.

In summary, compared to traditional target detection models, YOLO series models, and RT-DETR series models, the PHRF-RTDETR model demonstrates superior overall performance in weed detection for upland rice, which balances both detection accuracy and lightweight efficiency.

### 3.8 Visual analysis of PHRF-RTDETR model detection performance

**Figure 16:**
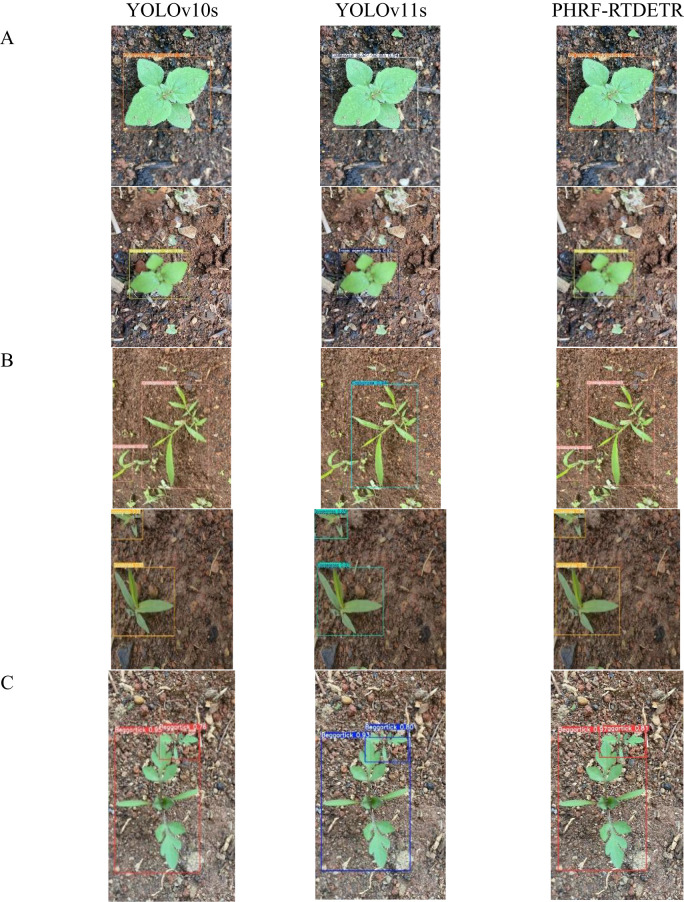
Detection graphs of different scenarios. **(A)** Single target. **(B)** Multiple target. **(C)** Occlusion.

The weed environment in upland fields is characterized by its non-structural property. Weed detection was performed in upland rice fields across different scenarios, including single-target, multi-target, and occluded conditions using YOLOv10s, YOLOv11s, and PHRF-RTDETR models. The corresponding results are illustrated in **Figure 16**.

In the single-target scenario, all three models achieved high confidence scores of 0.92 or above for weed detection, with no significant differences observed among them. However, in multi-target scenarios, the detection superiority of PHRF-RTDETR became evident. Compared to PHRF-RTDETR, both YOLOv10s and YOLOv11s exhibited lower overall confidence scores in detecting weeds, and YOLOv11s additionally suffered from missed detections. In occlusion scenarios, PHRF-RTDETR demonstrated confidence scores of 0.97 and 0.81, respectively, outperforming those of both YOLOv10s and YOLOv11s.

In conclusion, through detection experiments across various scenarios, the PHRF-RTDETR model demonstrated well-pleasing performance in weed detection in upland fields. Whether in single-target, multi-target, or occluded scenarios, the PHRF-RTDETR model consistently and reliably detected weeds in upland fields.

## 4 Discussion

Wang K. et al. (2024). employed an improved YOLOv7 model to detect five common weed species in wheat fields, achieving a high precision rate of 97.7% and recall of 98.8%, which surpassed the performance metrics of our proposed model. However, their approach demands substantially greater computational resources, rendering it less suitable for deployment on resource-constrained field devices. Cai et al. (2023) performed weed segmentation analysis in pineapple fields using UAV-based imagery, achieving a mean pixel accuracy (MPA) of 88.66%. Their work establishes an important reference for developing adaptive spraying systems using drone platforms. While UAV-based herbicide application demonstrates superior operational efficiency, this approach becomes non-operational during adverse weather conditions. By contrast, our research provides robust technical support for ground-based spraying robots and mechanical weeding systems, which exhibit significantly better operational reliability under various weather conditions. Arsa et al. (2023) developed an encoder–decoder convolutional neural network with dual decoder branches for weed growth point detection, achieving a detection rate of 85.05%. This method provides satisfactory detection performance for laser-based weeding systems. However, the high equipment costs associated with laser weeding technology significantly increase the operational expenses, limiting its economic feasibility for widespread agricultural applications. In contrast, our proposed method offers a more cost-effective solution for both mechanical and spray-based weeding robots, maintaining competitive detection accuracy while substantially reducing the economic barriers to implementation. Fan et al. (2023) implemented an enhanced Faster R-CNN architecture for weed detection in cotton fields, achieving a remarkable mAP of 98.43%. They successfully deployed this model on a sprayer robot, demonstrating an effective spraying rate of 98.93% and an average coverage rate of target areas of 97.42%. The image acquisition height of their study was 30 to 60 cm, which achieved good weeding effect. In contrast to our study, the acquisition height was about 30 cm, which proved the rationality and applicability of the imaging height used in this paper for the ground spraying weeding robot. Wang Y. et al. (2024) developed an optimized dynamic coverage algorithm for selective mechanical weeding robots, enabling precision weeding operations within an 80-cm height range. By contrast, the imaging height condition of about 30 cm in our study is also suitable for the working range of the weeding actuator of the mechanical robot.

The proposed PHRF-RTDETR model generates detection outputs that provide intelligent spraying and mechanical weeding robots with precise identification and localization information. Based on these outputs, the decision-making module enables optimized path planning, allowing the robots to accurately execute herbicide application or physical removal within targeted grid cells containing weeds. Compared to drone spraying systems and laser-based weeding robots, our detection model demonstrates superior adaptability to adverse weather conditions while maintaining significant cost-effectiveness advantages for field operations.

However, this study has two main limitations that should be acknowledged. First, although the lightweight detection performance of PHRF-RTDETR has been thoroughly analyzed and validated, the model has not yet been deployed or tested on actual weeding equipment. Thus, its practical performance under real-world field conditions requires further verification. Second, the dataset used in this study only includes dominant weed species in upland rice field. Consequently, the detection performance for other weed types remains unexplored. This investigation focuses particularly on early-growth weed detection in low-density conditions, representing the most economically efficient treatment period, while high-density weed infestations in later growth stages have not been addressed. These aspects represent important directions for future research.

## 5 Conclusion

In this study, we developed a lightweight weed detection model for upland rice, named PHRF-RTDETR. Compared with the baseline RT-DETR model, PHRF-RTDETR achieved reductions of 59.3%, 53.7%, and 53.9% in FLOPs, Params, and Size, respectively, with values of 23.2 G, 9.2 M, and 17.8 MB. The P, R, mAP50, and mAP50:95 of PHRF-RTDETR showed little difference from the original RT-DETR, achieving 92%, 85.6%, 88.2%, and 76.6%, respectively. Overall, the PHRF-RTDETR model strikes a better balance between lightweight performance and weed detection accuracy for upland rice, which demonstrates significant potential for deployment in the detection modules of edge devices, offering valuable technical insights for the advancement of an intelligent weeding robot. Such technological advancement could significantly improve upland rice productivity, offering a sustainable solution to regional food security challenges.

Future research will focus on advancing the practical application of our model in smart agricultural weeding equipment while enhancing its generalization capabilities. Specifically, we will pursue the following directions: First, we will implement quantization of the PHRF-RTDETR model weight file followed by deployment on mobile edge computing platforms such as NVIDIA Jetson series Raspberry Pi series, Huawei Atlas 200, etc. Through integration with platform dynamics simulation, we will quantitatively evaluate the impact of large speed variations on imaging quality to optimize the corresponding parameters and configurations. Second, we will expand the image dataset to include greater diversity in weed species and density levels, enabling more robust performance in practical agricultural settings.

## Data Availability

The original contributions presented in the study are included in the article/supplementary material. Further inquiries can be directed to the corresponding author.
